# Assessment of Pain, Acceptance of Illness, Adjustment to Life, and Strategies of Coping with Illness among Patients with Gastric Cancer

**DOI:** 10.1007/s13187-019-01519-0

**Published:** 2019-04-11

**Authors:** Urszula Religioni, Aleksandra Czerw, Anna M. Badowska-Kozakiewicz, Andrzej Deptała

**Affiliations:** 1 Department of Economic and System Analyses, National Institute of Public Health – NIH, Warsaw, Poland; 2 Collegium of Business Administration, Warsaw School of Economics, Warsaw, Poland; 3 Department of Health Economics and Medical Law, Medical University of Warsaw, Warsaw, Poland; 4 Department of Biophysics and Human Physiology, Medical University of Warsaw, Warsaw, Poland; 5 Department of Oncology and Hematology, Central Clinical Hospital of the MSWiA in Warsaw, Warsaw, Poland; 6 Division of Cancer Prevention, Medical University of Warsaw, Warsaw, Poland

**Keywords:** Quality of life, Gastric cancer, Acceptance of illness, Pain

## Abstract

Gastric cancer is the fourth leading cause of deaths in Poland. The standard treatment for non-advanced gastric cancer is surgery, which significantly reduces the quality of life of patients. The objective of the study was to evaluate the strategy of coping with pain and its control, acceptance of illness, and adjustment to living with cancer in patients suffering from gastric cancer. The analysis of the impact of socio-economic factors on the above-mentioned problems was also analyzed. The study was conducted among 93 patients diagnosed with gastric cancer, treated on an outpatient basis at the Oncology Center—Maria Skłodowska—Curie Institute in Warsaw in 2017–2018. The PAPI (paper and pencil interview) technique was used. The questionnaire interview included metric questions (socio-economic variables) and four psychometric tests: BPCQ (the Beliefs about Pain Control Questionnaire), CSQ (the Pain Coping Strategies Questionnaire), AIS (Acceptance of Illness Scale), and Mini-MAC (Mental Adjustment to Cancer) test. In the area of pain control, patients with gastric cancer assign the greatest role to internal factors (M = 16.34, SD = 4.93), although women obtained the highest value in the impact of physicians. In the area of coping with pain, patients most likely select the strategy of praying/hoping (M = 22.19, SD = 9.36). The mean value of acceptance of illness for patients with gastric cancer is M = 24.02, SD = 7.69, and it is not conditioned by any socio-economic variable. In the area of mental adjustment to illness, the highest values were obtained by positive reevaluation (M = 20.73, SD = 3.35) and fighting spirit (M = 20.68, SD = 3.98). Patients with gastric cancer control pain mainly through internal factors. The most frequently chosen strategy for coping with pain is praying/hoping, and positive reevaluation prevails in the field of mental adjustment. The results point to specific factors that can affect the patient’s pain, quality of life, and treatment outcomes. Knowing the diversity of these factors, it is possible to plan specific psychotherapeutic activities for specific groups of people that could be a supplement to the standard treatment process.

## Introduction

Differences in the incidence of gastric cancer in the world significantly vary between populations. The highest incidence is observed in the eastern part of Asia (over 40/100,000 people), in Central America (30/100,000 people), in Eastern Europe (around 25/100,000 people), and in South America (20/100,000 people) [1]. In Poland, gastric cancer is the fifth most frequent cancer among men and the ninth among women, and in terms of cancer mortality, it ranks the fourth cause of death among men and the sixth cause of death among women [2]. In the world, gastric cancer is the third most common cause of death due to cancer [3]. Currently, the standardized rate for developing gastric cancer in Poland is 7.7/100,000 people. In 1980, it was 16.7. The standardized death rate for gastric cancer in Poland is in total 7.4/100,000 people—for comparison, in 1980, it was 19.7; and in 1965, 30.6 [4, 5].

In Poland, the incidence of gastric cancer among men is higher than in most countries of the European Union, while among women in Poland, gastric cancer occurs less often than among women in most of these countries. However, in both men and women, mortality rates are higher in Poland than in other European Union countries (in men by about 25%, in women by about 10%).

The risk of gastric cancer increases with age, significantly higher in men than in women (about 2–3 times). The highest incidence of gastric cancer in Poland occurs among a population over 50 years of age. The 5-year survival rate among patients with cancer is 17.6%, and it is higher in women (19.8%) than in men (16.4%) [5]. The standard in the treatment of non-advanced gastric cancer is surgery—gastrectomy with lymphadenectomy of regional lymph nodes [6]. Due to the diagnosis of gastric cancer in a more advanced stage, in most cases, perioperative or complementary chemotherapy is necessary, which further affects the quality of life of patients [4, 7].

In the assessment of the health-related quality of life, the influence of psychological factors should also be emphasized. Literature indicates in particular such psychological factors as patients’ attitude towards disease, the ability to cope with stress and emotions, acceptance of the disease, or mental adjustment to the disease [8–10]. The adopted attitude towards pain and disease not only affects the quality of life but also may even determine the final effects of patient therapy. The research also indicates the impact of positive attitude, willingness to fight or the level of fear and anxiety on the effectiveness of treatment [11–13].

### Objective

The objective of the study was to evaluate the strategy of coping with pain, pain control, acceptance of illness, and adjustment to life with cancer in patients suffering from gastric cancer. The analysis also included the impact of socio-economic factors (gender, age, education, professional status, income, place of residence) and chemotherapeutic treatment on the results obtained in psychometric tests.

## Material and Methods

The study was conducted among 93 patients diagnosed with gastric cancer, who underwent radical resection and chemotherapy, being under outpatient care at the Oncology Center—Maria Skłodowska—Curie Institute in Warsaw in 2017–2018. The PAPI (paper and pencil interview) technique was used. The questionnaire interview included metric questions (socio-economic variables) and four psychometric tests:

1. The Beliefs about Pain Control Questionnaire (BPCQ), designed to examine people suffering from pain [14]. It consists of 13 statements that are part of three dimensions that measure the strength of individual beliefs about controlling pain personally (internal factors), through the influence of physicians (powerful others), or by chance events. Each of the statements is rated by the respondent on a scale of 1 to 6, where 1 means “no, I totally disagree” and 6 “yes, I totally agree.” For each BPCQ test dimension, the sum of the results is calculated separately on the basis of the sum of points obtained in individual statements. The higher the score, the stronger the effect of a given dimension on the patient’s pain control.
2. The Pain Coping Strategies Questionnaire (CSQ), used to examine people who complain of pain [15]. The questionnaire consists of 42 statements, and individual statements are evaluated using a scale from 0 to 6. The obtained points reflect six cognitive strategies (diverting attention, reinterpreting pain sensations, catastrophizing, ignoring pain, praying/hoping, coping self-statements) and one behavioral strategy (increased behavioral activity). For each strategy, the result is calculated, ranging from 0 to 36 points. The higher the score, the more importance is attributed to a given factor in the process of dealing with pain.
3. Approval Illness Scale (AIS), measuring the level of adjustment to illness [16]. The AIS contains eight statements referring to the negative consequences of poor health. These consequences are based on the recognition of limitations imposed by the disease, feelings of dependence on others, lowered self-esteem, and lack of self-sufficiency. Each of the eight statements used in AIS is assigned a scale from 1 to 5, where 1 means “I definitely agree,” and 5 “I strongly disagree.” The patient may score between 8 and 40 points, which will reflect the degree of illness acceptance.
4. Mental Adjustment to Cancer (Mini-MAC), measuring the level of mental adjustment to cancer [17]. The questionnaire consists of 29 statements indicating 4 ways of coping with disease: anxious preoccupation, fighting spirit, helplessness-hopelessness, and positive reevaluation. While anxious preoccupation and helplessness-hopelessness form the passive (destructive) style of coping with the disease, fighting spirit, and positive reevaluation refer to the active (constructive) way of coping. Each statement of the mini-MAC is assessed by the respondent on a four-scale ranging from 1 (definitely not) to 4 (definitely yes). The points in each strategy are calculated separately based on the total scores obtained in specific statements, and the final result may be from 7 to 28 points. The higher the score, the stronger the severity of behaviors characteristic for a given strategy of coping with the disease.

The obtained results were subjected to statistical analysis using Student’s *t* test for independent samples, one-way analysis of variance, and Pearson’s r correlation. The assumed level of statistical significance is *p* < 0.05.

## Results

The study involved patients aged 23–84 (M = 59.98, SD = 14.25), including 45 (48.4%) women aged 23–84 (M = 57.67, SD = 14.17) and 48 (51.6%) men aged 28–81 (M = 62.15, SD = 14.14).

Among the studied group of patients, 33 (35.5%) have primary/vocational education, 33 (35.5%) have secondary, and 27 (29.0%) have higher education. In towns with a population of up to 100,000 live 43 (46.2%) patients, and in cities with a population of over 100,000 live 50 (53.8%) patients. More than half of patients have a monthly net income of up to PLN 1500 (48 patients, 51.6%), and 45 (48.4%) patients indicated that they achieved income over PLN 1500.00. There were 36 people (38.7%) working in the study group of patients, and 57 (61.3%) were pensioners.

### Pain Control

The Beliefs about Pain Control Questionnaire, measuring the strength of beliefs about pain control by means of 13 statements, characterizes patients as individuals controlling pain in person (through internal factors), the influence of physicians (external factors, the strength of others), or by random events.

Patients with diagnosed gastric cancer control pain primarily due to internal factors (M = 16.34, SD = 4.93), least frequently by random events (M = 15.33, SD = 3.96), although the values of all dimensions are similar (Table 1).

**Table 1:** BPCQ results for patients with gastric cancer

BPCQ dimension	Mean (M)	Standard deviation (SD)
Internal factors	16.34	4.93
Impact of physicians	16.05	4.14
Random events	15.33	3.96

Pain control, mostly within internal factors, was the domain of men (M = 16.96, SD = 4.87). Women obtained the highest value in the impact of doctors (M = 15.87, SD = 4.51), but these differences proved to be statistically insignificant (*p* > 0.05). The age of patients positively correlated with locating pain control in random events (*r* = 0.246)—older patients attributed a greater role to this dimension.

Patients with primary/vocational education most often control pain through the impact of physicians (M = 16.97, SD = 4.39), and patients with secondary and higher education attribute the greatest role to internal factors (M = 16.09, SD = 5.08, respectively for people with secondary education and M = 17.52, SD = 4.52 for people with higher education). A similar relationship was observed depending on the occupational status of patients; working people (M = 17.08, SD = 4.83) attribute a greater role to internal factors than pensioners (M = 15.81, SD = 5.07). In both cases, however, these differences are not statistically significant (*p* > 0.05). Similarly, there were no significant differences depending on the place of residence of the patients (a city of up to 100,000 residents and a city of over 100,000 residents) (*p* > 0.05).

Patients with higher income (over PLN 1500 per person in a household) achieved a much higher score in internal factors than patients with income below PLN 1500 per person in a household, but the statistically significant difference covers only the dimension of random events (*p* = 0.035). The mean value of the severity of location of pain control in random events was lower in the group of people with higher income (M = 14.44, SD = 4.02) compared with the group with income below PLN 1500 per person in a household (M = 16.17, SD = 3.75).

Internal pain control was assessed considerably higher by people who underwent chemotherapy in the last year (M = 17.44, SD = 4.87) compared with people who did not receive chemotherapeutic treatment (M = 15.3, SD = 4.81) (*p* = 0.037).

### Strategies for Coping with Pain

Patients with gastric cancer usually cope with pain through praying/hoping (M = 22.19, SD = 9.36) and declaring coping (M = 21.08, SD = 7.93). The lowest-rated strategies were reevaluation of pain sensations (M = 12.65, SD = 7.98) and catastrophizing (M = 15.04, SD = 7.68) (Table 2).

**Table 2:** CSQ results for patients with gastric cancer

CSQ dimension	Mean (M)	Standard deviation (SD)
Distraction	19.94	7.99
Revaluation of pain sensations	12.65	7.98
Catastrophizing	15.04	7.68
Ignoring sensations	15.65	8.46
Praying/hoping	22.19	9.36
Declaring coping	21.08	7.93
Increased behavioral activity	20.22	7.64

The strategy of pain control most often declared by women was praying/hoping, and in men, it was declaring coping. These differences are not statistically significant (*p* > 0.05). A statistically significant negative correlation was found between the age of the patients and the reevaluation of pain sensations (*r* = − 0.245). Younger people more often chose a strategy for reevaluation of pain sensations than older people did.

Although praying/hoping was the most frequently chosen strategy by each group, divided into education (primary/vocational, secondary, higher), people with primary education obtained the highest score in this dimension (for primary/vocational education M = 23.42, SD = 9.82; for secondary education M = 20.82, SD = 9.33; and for higher education M = 22.37, SD = 8.95). However, these differences are not statistically significant (*p* > 0.05). The chosen strategy of coping with pain does not depend on the place of residence (*p* > 0.05).

People with higher income (over PLN 1500 per person in a household) achieved slightly lower results for the praying/hoping or catastrophizing dimension than persons with income below PLN 1500 per person in a household. People with higher income, however, had higher scores for the dimensions of declaring coping or increased behavioral activity, however, these differences are not statistically significant (*p* > 0.05).

A statistically significant difference was obtained in the scope of reevaluation of pain sensations depending on the professional status of the patients. The mean value obtained in the group of pensioners (M = 10.10, SD = 7.39) was statistically significantly lower than the mean value obtained in the working group (M = 15.28, SD = 8.12) (*p* = 0.003). Working people also obtained significantly higher scores in the dimensions of declaring coping and increased behavioral activity, but they were not statistically significant (*p* > 0.05).

Chemotherapy treatment did not significantly affect the results obtained by the patients (*p* > 0.05).

### Acceptance of Illness

The general acceptance of illness on the AIS obtained by patients with gastric cancer was M = 24.02 with a standard deviation of SD = 7.69.

The mean value of acceptance of illness in the group of women was M = 23.00 with a SD = 7.35 and was close to the mean value obtained in the group of men, which was M = 24.98 with a SD = 7.95. Acceptance of illness did not significantly correlate statistically with the age of patients (*p* > 0.05).

The mean value of acceptance of illness in the group of people with primary or vocational education was M = 23.33 (SD = 8.26), in the group of people with secondary education was M = 24.55 (SD = 7.97), and in the group of people with higher education was M = 24.22 (SD = 6.79). Based on the value of the one-way analysis of variance, it was found that the differences were statistically insignificant (*p* > 0.05).

The mean value of acceptance of illness in the group of people living in towns with a population of up to 100,000 was M = 25.44 (SD = 7.70), and it was close to the mean value obtained in the group of people who lived in cities with a population of over 100,000 of M = 22.80 (SD = 7.54). Based on the Student’s *t* test value for independent samples, it was found that the difference obtained was statistically insignificant (*p* > 0.05).

The mean value of acceptance of illness in the group of people with net income of up to PLN 1500 was M = 23.77 (SD = 7.60), and it was close to the mean value obtained in the group of people with income above PLN 1500, amounting to M = 24.29 (SD = 7.86). Based on the Student’s *t* test value for independent samples, it was found that the difference received was irrelevant (*p* > 0.05).

The mean value of acceptance of illness in the working group was M = 25.22 (SD = 7.43), and it was close to the mean value obtained in the group of pensioners amounting to M = 23.27 (SD = 8.10), which was also a result that was not statistically significant (*p* > 0.05).

Chemotherapy in the last year also did not affect acceptance of illness among the patients (*p* > 0.05). The mean value of acceptance of illness in the group of people who underwent chemotherapy was M = 23.71 (SD = 7.18), and in the group of people who did not undergo chemotherapeutic treatment, it was M = 24.31 (SD = 8.20).

### Mental Adjustment to Illness

The patients with gastric cancer obtained the highest result in the dimension of positive reevaluation (M = 20.73, SD = 3.35) in the Mini-MAC test, measuring mental adjustment to illness. A similar value characterized fighting spirit (M = 20.68, SD = 3.98). The lowest result was obtained in the dimension helplessness-hopelessness (M = 14.62, SD = 4.11) (Table 3).

**Table 3:** Mini-MAC test results for patients with gastric cancer

Mini-MAC dimension	Mean (M)	Standard deviation (SD)
Anxiety	17.83	4.23
Fighting spirit	20.68	3.98
Helplessness-hopelessness	14.62	4.11
Positive revaluation	20.73	3.35

Among women, the dominant dimension of mental adjustment to illness was a positive reevaluation, and among men, fighting spirit. It was noticed that women obtained higher results in the dimensions of anxiety (M = 18.24, SD = 4.50) and helplessness-hopelessness (M = 15.07, SD = 4.16) in comparison with men (the mean for the dimension of anxiety for men was M = 17.44, SD = 3.97; and for the dimension helplessness-hopelessness M = 14.21, SD = 4.07), but they are not statistically significant. (*p* > 0.05).

Age and place of residence do not determine the way of mental adjustment to illness (in both cases *p* > 0.05). Similarly, education does not significantly affect the differences in patients’ adjustment to illness, although the mean higher values of anxiety in people with secondary education (M = 18.64, SD = 4.08) and higher education (M = 18.04, SD = 4.16) compared primary/vocational education (M = 16.85, SD = 4.37) were observed.

Persons with lower income (below PLN 1500 per person in a household) achieved a higher result in terms of hopelessness and hopelessness (M = 15.25, SD = 4.29) than people with higher income (M = 13.96, SD = 3), but the differences were not statistically significant (*p* > 0.05).

Comparing working people and pensioners, it was noted that the mean value of positive reevaluation was higher in the group of pensioners (M = 21.18, SD = 3.18) than that among the working population (M = 19.72, SD = 3.49) (*p* = 0.048).

Chemotherapy did not affect the results obtained by the patients in terms of mental adjustment to illness (*p* > 0.05).

## Discussion

The diagnosis of cancer entails long-term emotional consequences for patients, associated with long-term treatment, reduced quality of life, and often the need to deal with the side effects of the treatment. These factors affect the patient’s social life and ability to work. Physical symptoms of the disease are often associated with various mental disorders. However, depending on the particular types of behavior, as well as previous experiences, the attitude towards the disease, including disease acceptance, may vary considerably.

Studies show that the quality of life of patients is deteriorating already at the time of receiving the diagnosis, which is often associated with dismay, anxiety, and a sense of powerlessness. However, adjustment to cancer and the associated problems, acceptance of illness, and satisfaction from various areas of life may result in the quality of life of patients with cancer returning to a relatively good level [18, 19]. However, some sources indicate that depression symptoms often occur among cancer patients and that cancer patients are characterized by a significantly higher level of anxiety [20].

Numerous studies show that gastric cancer patients notice a significant decrease in their quality of life in the first year after surgery, which may still be associated with a relatively short time after receiving the diagnosis and thus with continued anxiety. After this time, the quality of life assessed by patients increases systematically. Usually, the level of quality of life in the fifth year after surgery is comparable with that before cancer [21–23]. Shortly after surgery, gastric cancer patients assess their quality of life as being the lowest—even 70% of patients indicate a significant deterioration of the quality of life [21]. However, studies conducted by Lee S.S. et al. indicate that 5 years after surgery, patients with gastric cancer still show a lower quality of life compared with the control group [24]. The most common ailments related to the treatment affecting the quality of life of patients with gastric cancer include a feeling of post-meal fullness, diarrhea, difficulty swallowing, heartburn, and the need to comply with a restrictive diet [22].

Literature indicates that the quality of life assessed by patients is influenced by both external and internal factors. External factors include the level of financial stabilization, housing situation, the level of education, maintaining social contacts, or the type of treatment undertaken. Internal factors, which may contribute to the increase of the quality of life assessed by patients, are internal resources, including coping with stress and strategies for coping with cancer or personality traits (optimism) [25, 26].

The study shows that patients with gastric cancer control pain mainly through internal factors (M = 16.34, SD = 4.93). This was influenced by undergoing chemotherapy within the past 12 months. Patients who underwent chemotherapy obtained a higher score in the dimension of pain control through internal factors. As a comparison, in the case of another cancer of the digestive tract—colorectal cancer—patients also attributed the highest impact in controlling pain to internal factors (M = 17.36, SD = 5.47) [27].

The most common way of dealing with pain in patients with gastric cancer is praying/hoping (M = 22.19, SD = 9.36) and declaring coping (M = 21.08, SD = 7.93). In the case of colorectal cancer patients, the predominant strategies for coping with pain are declaring coping (M = 21.86, SD = 9.43) and increased behavioral activity (M = 21.42, SD = 9.16) [27]. The lowest-rated strategy in the case of patients with gastric cancer was the reevaluation of pain sensations (M = 12.65, SD = 7.98), whereas for patients with colorectal cancer in the study conducted by Czerw A. et al., the lowest result was obtained by patients in the dimension of catastrophizing (M = 10.04, SD = 7.75) [21]. In our study conducted on patients with gastric cancer, the strategy of catastrophizing reached a significantly higher value of M = 15.04 (SD = 7.69).

In the study by Kózka M. et al. conducted among 102 cancer patients hospitalized at oncology, radiotherapy and chemotherapy departments indicated that patients usually cope with pain through praying/hoping, and less often by declaring coping. The overall mean acceptance of illness for cancer patients on the AIS was M = 24.9 (SD = 6.35) [28]. In our study, patients with gastric cancer obtained a similar mean result of acceptance of illness (M = 24.02, SD = 7.69).

The study conducted by Kozak G. among patients with diagnosed gastric cancer, cancer of reproductive organs, pancreatic cancer, colorectal cancer, prostate cancer, staying in palliative care in the hospital or at home indicates that, in patients with gastric cancer, the degree of the intensity of fighting spirit grows with an increase of acceptance of the illness, whereas the level of anxiety and helplessness-hopelessness decreases. The average level of acceptance of illness at the AIS for patients with gastric cancer was significantly lower than in our study (M = 16.87, SD = 5.59). As a comparison, in the study conducted by Kozak G., patients with pancreatic cancer reached the mean value of M = 18.23 (SD = 9.13), and with colorectal cancer M = 16.58 (SD = 7.42) [29]. Studies conducted by Kapela I. et al. also indicate that patients with colorectal cancer accept their illness significantly better (M = 28.4) [18] compared with patients with gastric cancer who were included in our study. A similar result of the level of acceptance of the illness among people with colorectal cancer, showing that those patients are better able to accept their illness than those suffering from gastric cancer, is indicated by Religioni U. et al. [30]. In the case of colorectal cancer, gender, marital status, occupational status, place of residence, or duration of cancer do not affect the results of acceptance of the illness [18]. Our study also indicates that the acceptance of the illness does not depend on the studied socio-economic variables.

In our study, patients with gastric cancer most often chose the strategy of positive reevaluation when it came to the mental adjustment to illness (M = 20.73, SD = 3.35). However, studies conducted by Juczyński Z. indicate that anxiety and hopelessness-helplessness reach high values in patients with gastric cancer and pancreatic cancer [17]. Similar results were demonstrated in studied conducted by Kozak G., where anxiety achieved the average result of M = 22.84 (SD = 5.52), and helplessness-hopelessness M = 20.98 (SD = 5.68) [29]. In our study, anxiety achieved a value of M = 17.83 (SD = 4.23), and hopelessness-helplessness M = 14.62 (SD = 4.11).

In the case of patients with colorectal cancer, on the Mini-MAC scale, the constructive style dominates (M = 46.4 in comparison with M = 28.5 for the destructive style) with an advantage of fighting spirit (M = 23.9) and positive reevaluation (M = 22.5) [18]. Fighting spirit also dominates in patients with colorectal cancer in the study conducted by Czerw A. (M = 23.43) [27]. In the study conducted by Kapela I. et al., a certain relationship between the results on the Mini-MAC scale and age was observed [18]. In our study on patients with gastric cancer, pensioners obtained a higher mean result for positive reevaluation than working people.

Similarly to the researches of other authors, the conducted study indicates that patients choose different types of adaptations to the disease or strategies for coping with pain. The adopted strategy significantly affects the quality of life of patients as well as the effectiveness of treatment [31–33]. Due to the perceived complaints, including pain associated with cancer, the acceptance of the disease and the adopted strategy for coping with pain are areas that pose great difficulties for patients.

The awareness of the diversity of factors contributing to the selection of a particular strategy of adjustment to cancer makes it is possible to conduct specific psychotherapeutic activities for specific groups of patients that could supplement the standard treatment process. Constructive strategies for adapting to the disease should be an important element of education for both psychologists and doctors as well as for the patients themselves.

There are limitations to every study design. In this study, tools measuring the time from the diagnosis of the disease to the date of testing were not used. It can be assumed that the longer the duration of the disease, the greater the acceptance of the disease. Nevertheless, the analysis of other studies published in this field suggests that our method is comparable with other research.

## Conclusions

1. Patients with gastric cancer control pain mainly through internal factors, although women have the highest mean in terms of the impact of physicians.
2. Among the strategies for coping with pain, patients with gastric cancer most often indicated praying/hoping and declaring coping.
3. The level of acceptance of illness among patients with gastric cancer reached the average result, and none of the variables examined significantly affected the level of acceptance of illness among patients.
4. In terms of mental adjustment to illness, patients most often chose the style of positive reevaluation.
5. The awareness of the level of disease acceptance or the strategy of coping with pain by specific groups of patients makes it possible to plan special psychotherapeutic activities for specific groups of patients.
6. The significantly higher incidence of stomach cancer among men in Poland indicates that psychotherapeutic support in the fight against cancer should be addressed specifically to this group of people.
